# The hypoxia-activated prodrug evofosfamide in combination with multiple regimens of radiotherapy

**DOI:** 10.18632/oncotarget.15784

**Published:** 2017-02-28

**Authors:** Katarzyna J. Nytko, Ivo Grgic, Sabine Bender, Janosch Ott, Matthias Guckenberger, Oliver Riesterer, Martin Pruschy

**Affiliations:** ^1^ Laboratory for Applied Radiobiology, Department of Radiation Oncology, University Hospital Zurich, Zurich, Switzerland; ^2^ Clinical Research Priority Program “Tumor Oxygenation”, Zurich, Switzerland; ^3^ Department of Radiation Oncology, University Hospital Zurich, Zurich, Switzerland

**Keywords:** evofosfamide, TH-302, hypoxia-activated prodrug, ionizing radiation, P450 oxidoreductase

## Abstract

The promising treatment combination of ionizing radiation (IR) with a hypoxia-activated prodrug (HAP) is based on biological cooperation. Here we investigated the hypoxia-activated prodrug evofosfamide in combination with different treatment regimens of IR against lung A549- and head&neck UT-SCC-14-derived tumor xenografts. DNA damage-related endpoints and clonogenic cell survival of A549 and UT-SCC-14 carcinoma cells were probed under normoxia and hypoxia.

Evofosfamide (TH-302) induced DNA-damage and a dose-dependent antiproliferative response in A549 cells on cellular pretreatment under hypoxia, and supra-additively reduced clonogenic survival in combination with IR. Concomitant treatment of A549-derived tumor xenografts with evofosfamide and fractionated irradiation induced the strongest treatment response in comparison to the corresponding neoadjuvant and adjuvant regimens. Adjuvant evofosfamide was more potent than concomitant and neoadjuvant evofosfamide when combined with a single high dose of IR. Hypoxic UT-SCC-14 cells and tumor xenografts thereof were resistant to evofosfamide alone and in combination with IR, most probably due to reduced P450 oxidoreductase expression, which might act as major predictive determinant of sensitivity to HAPs.

In conclusion, evofosfamide with IR is a potent combined treatment modality against hypoxic tumors. However, the efficacy and the therapeutic outcome of this combined treatment modality is, as indicated here in preclinical tumor models, dependent on scheduling parameters and tumor type, which is most probably related to the status of respective HAP-activating oxidoreductases. Further biomarker development is necessary for the launch of successful clinical trials.

## INTRODUCTION

Radiotherapy, along with surgery and chemotherapy is one of the major treatment options for solid tumors. However, solid tumors are often radiation resistant due to tumor hypoxia, which thereby represents a major clinical challenge. Several strategies have been developped during the last decades to overcome the hurdle of tumor hypoxia for successful radiotherapy [1–4]. One of these concepts is based on biological cooperation, which refers to strategies that target distinct cell populations, or employ different mechanisms for cell killing. The combination of ionizing radiation (IR) with a Hypoxia-Activated Prodrug (HAP), targeting hypoxic tumor cells and thereby complementing the effect of IR in well-oxygenated cells, nicely represents the concept of biological cooperation [5]. Originally, nitrobenzenes, followed by the nitroimidazoles (misonidazole, etanidazole, pimonidazole) [6], were proposed to act as oxygen mimetic agents and to generate together with short-lived IR-induced free DNA radicals cytotoxic DNA strand breaks [7, 8]. Unfortunately, and despite the clarity of the concept, severe toxicities of these early generation compounds have contributed that these hypoxic radiosensitizers did not find their recognition in the clinical routine. Nevertheless these findings paved the way for the generation of hypoxia-selective bioreductive prodrugs, which are activated by enzymatic reduction in hypoxic tissues [9].

This risk of severe side-effects is reduced with HAPs that are less toxic and especially with compounds that are only activated under severe hypoxia. At the same time such prodrugs should release a diffusible, active cytotoxic agent, not only to kill the most hypoxic tumor cells, but also to induce a bystander effect thereby killing tumor cells of intermediate levels of tumor hypoxia. The 2-nitroimidazole-conjugated bromo-isophosphoramide mustard (Br-IPM) evofosfamide (TH-302) represents a prototype of such a novel generation HAP. Evofosfamide is currently the most advanced compound in clinical trials of the new generation of bioreductive cytotoxins [10–12].

The development of linear accelerator technology for the precise delivery of radiotherapy has nowadays reached a level of dose conformity to the tumor that allows the application of high dose fractions (even > 10 Gy) to small tumors with very steep dose gradients. Fractionated application of daily doses of IR exploits reoxygenation of hypoxic tumor regions in between fractions and thereby overcomes the hypoxic challenge as part of an iterative process. On the other hand single high doses of IR or a hypofractionated treatment regimen requires other means to improve its efficacy and to control a hypoxic tumor e.g. by the combined treatment modality with a HAP [13, 14].

Here we investigated the potency of evofosfamide in combination with fractionated and single high-dose of IR and tested these regimens in three settings (neoadjuvant, concomitant and adjuvant) routinely applied in clinical practice. Furthermore, the influence of treatment conditions linked to the individual geno- and phenotype of the tumor, including the status of the HAP-activating oxidoreductases, DNA-damage repair machineries and the hypoxic burden, was analyzed.

## RESULTS

### Evofosfamide in combination with IR *in vivo*

**Figure 1:**
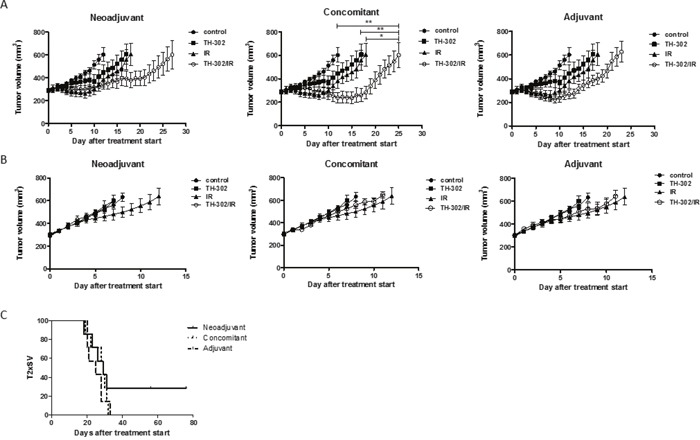
Treatment response to evofosfamide and fractionated irradiation *in vivo* Tumor growth delay of A549-derived **(A)** and UT-SCC-14-derived **(B)** tumor xenografts in response to different treatment schedules of the combined treatment modality with evofosfamide (50 mg/kg, Q3Dx5) and fractionated irradiation (3×2 Gy). Control mice were treated i.p. with saline. Neoadjuvant (left panel), concomitant (middle panel), and adjuvant (right panel) regimens are shown. Neoadjuvant (left panel), with evofosfamide given on days 1-12 followed by fractionated irradiation; concomitant (middle panel), with evofosfamide given on days 1-12 and IR on days 5-7; adjuvant (right panel), IR given on days 0-2 (fractionated) followed by treatment with evofosfamide. 7-8 mice per treatment groups were used. Error bars represent SEM. **(C)** Kaplan-Meier curves for A549-derived tumors reaching 600mm^3^ tumor volume.

The combined treatment modality of IR with an HAP is based on biological cooperation. However, the most effective scheduling of the two modalities is not predictable. We therefore probed three different treatment schedules (neoadjuvant, concomitant and adjuvant) of a minimally fractionated irradiation regimen (3×2 Gy on 3 consecutive days) and evofosfamide (50 mg/kg, Q3Dx5) in a lung adenocarcinoma (A549) and a head&neck squamous cell carcinoma (UT-SCC-14) tumor xenograft model. The dosage and schedule of evofosfamide was defined based on previous preclinical reports when used as part of a combined treatment modality [15] and closely mimic the settings used in clinical practice. 2 Gy per fraction of IR corresponds to the dose/fraction used as part of a clinical fractionated radiotherapy treatment regimen. Both tumor models were previously characterized to develop tumors with an intermediate hypoxic tumor fraction [16, 17]. Tumor hypoxia was also confirmed by pimonidazole staining (Supplementary Figure 1). Treatment was initiated when tumors reached a volume of 300 mm^3^ (+/− 10%). Treatment of A549-derived tumor xenografts with evofosfamide in combination with fractionated irradiation resulted in a strongly enhanced treatment response when compared to treatment with evofosfamide and irradiation alone (Figure 1A). The concomitant schedule induced the strongest tumor growth delay (*P*<0.05 for evofosfamide plus IR versus each monotherapy), however, no statistically significant differences in between the three combined treatment regimens could be determined. Interestingly, evofosfamide alone did not reduce tumor growth of HNSCC UT-SCC-14 xenografts and did not enhance the growth inhibitory effect of fractionated irradiation as part of a combined treatment modality (Figure 1B). These results suggest that the response to evofosfamide and IR is highly dependent on the tumor type.

**Figure 2:**
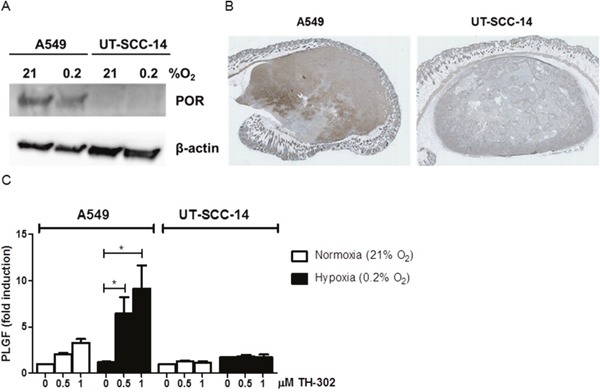
Differential POR- and PLGF-levels in A549 and UT-SCC-14 tumors **(A)** Protein levels of cytochrome P450 oxidoreductase (POR) in A549 and UT-SCC-14 cells incubated under normoxia (21% O_2_) and hypoxia (0.2% O_2_, 24 hours) as determined by western blotting. **(B)** Staining of A549 (left) and UT-SCC-14 (right)-derived tumor xenografts sections with anti–POR antibodies. **(C)** Levels of secreted PLGF in A549 and UT-SCC-14 conditioned medium in normoxia (21% O_2_) and hypoxia (0.2% O_2_, 24 hours) as determined using Bio-plex assay. Data are shown as fold induction over non-treated normoxic samples in three independent experiments, error bars represent SEM.

Due to the differential treatment response in the two tumor models *in vivo*, the effect of evofosfamide was also determined *in vitro* with defined hypoxic conditions (0.2% O_2_). Interestingly, A549 cells were also more sensitive than UT-SCC-14 to increasing concentrations of evofosfamide (Supplementary Figure 2). The cytochrome P450 oxidoreductase (POR) has previously been identified as major determinant for the sensitivity of hypoxia-activated prodrugs [18, 19]. Therefore, the expression level of POR was determined on the cellular and tumor level by western blotting and immunohistochemistry, respectively. The POR expression level was strongly reduced in UT-SCC-14 cells and UT-SCC-14-derived tumors in comparison to A549 cells and tumors derived thereof (Figure 2A, 2B). This is most probably the cause for evofosfamide-resistance against the head&neck tumor model used in this study. Furthermore, transient downregulation of POR in A549 cells with POR-directed siRNA resulted in reduced sensitivity to evofosfamide in these cells relative to control siLUC-transfected A549 cells (Supplementary Figure 3), reinforcing the role of POR for evofosfamide sensitivity. Despite several attempts, we could not perform the opposite experimental approach to overexpress POR in UTSCC-14 cells. These cells did always undergo cell death upon genetic manipulation alone.

To further analyze the differential treatment response in between A549 and UT-SCC-14-derived tumors, comprehensive analysis of hypoxia-related secreted factors was performed by Bio-plex analysis. Unfortunately, the levels of serum secreted factors in mice carrying tumor xenografts were below detection limits. Therefore, *in vitro* analysis of conditioned media derived from A549 and UT-SCC-14 cells was performed. The basal secretory levels of most factors analyzed were different in between the two cell lines (e.g. VEGF, IL-6, Osteopontin, sEGFR, TNFα) and did not change in response to evofosfamide treatment (Supplementary Figure 4). Interestingly, placental growth factor (PlGF) was strongly increased in A549 but not in UT-SCC-14 cells in response to evofosfamide, suggesting that an increase of PlGF might be used as an early response biomarker (Figure 2C).

**Figure 3:**
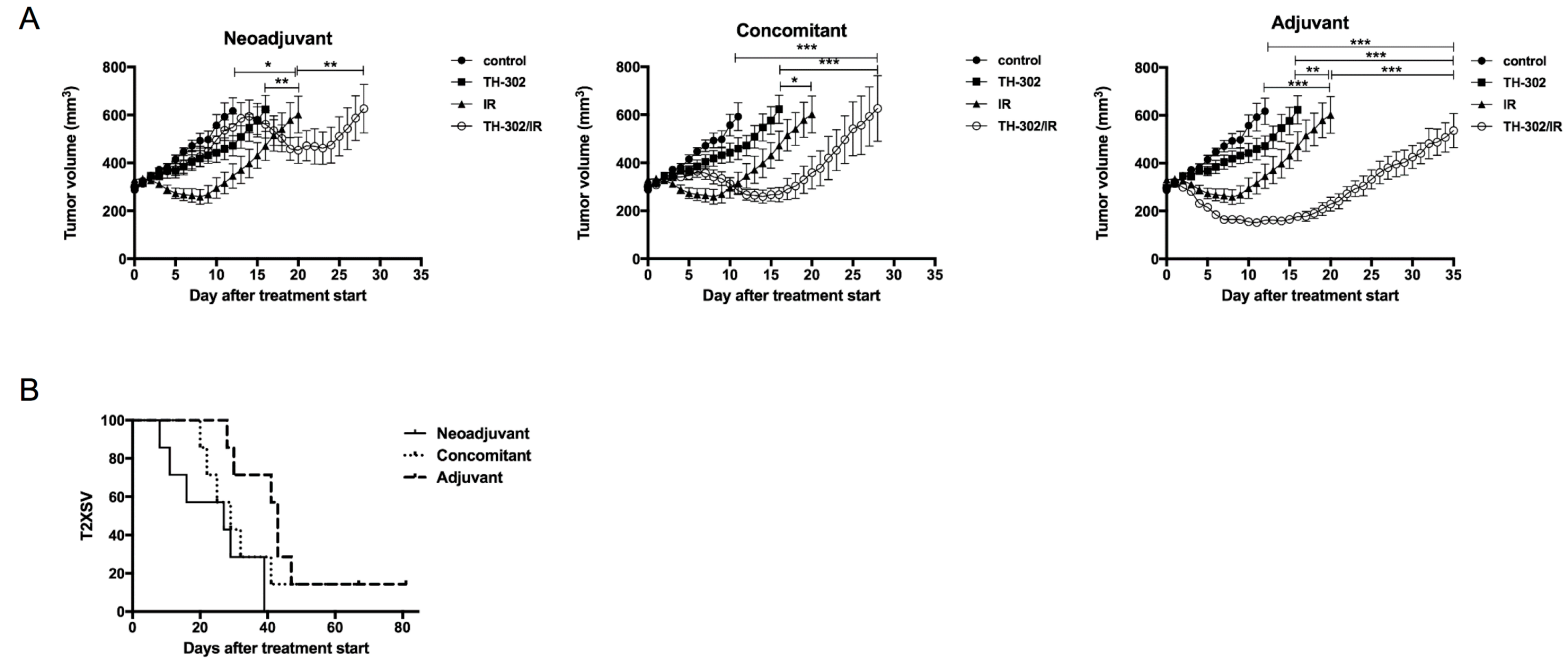
Treatment response to evofosfamide and single high-dose irradiation *in vivo* Tumor growth delay of A549-derived xenografts in immunocompromised mice in response to combined treatment with evofosfamide (50 mg/kg, Q3Dx5) and single high-dose IR (1×10 Gy). Control mice were treated i.p. with saline. **(A)** Neoadjuvant (left panel), concomitant (middle panel), and adjuvant (right panel) regimens are shown. Neoadjuvant (left panel), with evofosfamide given on days 1-12 followed by irradiation; concomitant (middle panel), with evofosfamide given on days 1-12 and IR on day 6; adjuvant (right panel), IR given on day 0 followed by treatment with evofosfamide. 7-14 mice per treatment groups were used. Error bars represent SEM. **(B)** Kaplan-Meier curves for tumors reaching 600mm^3^ tumor volume.

Next, the potency of evofosfamide was investigated in the evofosfamide-sensitive A549-derived tumor model as part of a combined treatment modality (neoadjuvant, concomitant, adjuvant) with a single high dose of IR (10 Gy). The adjuvant combined treatment modality was most effective and induced a strong tumor growth delay in comparison to evofosfamide and IR alone (*P*<0.05 for evofosfamide plus IR versus each monotherapy). Concomitant treatment with IR and evofosfamide only induced a partial additive tumor growth delay. On the other hand tumors almost doubled in size during the time period of neoadjuvant treatment with evofosfamide. Thereby tumors were irradiated at an increased tumor volume with a single high dose of IR, resulting in a reduced overall treatment response to the neoadjuvant combined treatment regimen (Figure 3A, 3B).

### Enhanced radiosensitivity upon evofosfamide treatment *in vitro*

**Figure 4:**
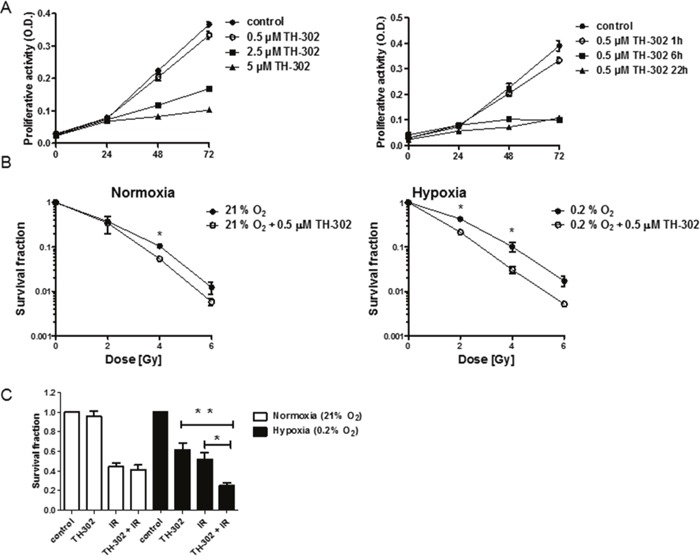
Treatment response to evofosfamide and irradiation *in vitro* **(A)** Proliferation of A549 lung adenocarcinoma cells in response to increasing doses of evofosfamide. Cells were pre-incubated in hypoxia (0.2% O_2_) for 23 hours, followed by treatment with increasing concentrations of evofosfamide under hypoxic conditions for 1 hour (left panel). Proliferation of A549 cells pre-incubated for 23, 18 and 2h in hypoxia (0.2% O_2_), followed by treatment with evofosfamide (0.5 μM) under hypoxia for 1, 6 and 22 hours, respectively (right panel). The proliferative activity of reoxygenated cells was monitored over 72 hours. **(B)** To determine time-dependent effects of evofosfamide, cells were incubated for 23, 18 and 2h in hypoxia (0.2% O_2_), followed by treatment with evofosfamide (0.5 μM) for 1, 6 and 22 hours, respectively. The proliferative activity of reoxygenated cells was monitored over 72 hours. **(B)** Clonogenic cell survival assay of A549 cells treated with 0.5 μM evofosfamide under normoxic (21% O_2_) and hypoxic (0.2% O_2_) conditions for 4 hours. Following reoxygenation, cells were irradiated with increasing doses of IR. **(C)** Clonogenic survival assay of lung carcinoma A549 cells irradiated with 2 Gy and treated thereafter with evofosfamide (0.5 μM) under normoxic (21% O_2_) and hypoxic (0.2% O_2_) conditions for 4 hours (adjuvant setting); Error bars represent SEM.

*In vitro* experiments with A549 cells demonstrated a dose- and hypoxia incubation time-dependent antiproliferative effect of evofosfamide (Figure 4A). To determine cancer cell clonogenicity, A549 cells were incubated with evofosfamide (0.5 μM) for 4 hours under hypoxia (0.2% O_2_) and normoxia, respectively, followed by irradiation under reoxygenated conditions. Combined treatment of A549 cells with evofosfamide and increasing doses of IR resulted in a strong, supra-additive reduction of clonogenicity when cells were preincubated with evofosfamide under hypoxic conditions in comparison to preincubation under normoxic conditions (DEF_0.1_=1.44+/−0.07 vs DEF_0.1_ of 1.16 +/− 0.07 respectively, and DEF_0.37_=1.72+/−0.12 vs DEF_0.37_=1.23+/−0.24, respectively) (Figure 4B).

The adjuvant schedule of evofosfamide in combination with IR resulted in a strong tumor growth delay *in vivo*. Therefore a reversed schedule with irradiation of cells (2 Gy) followed by incubation with evofosfamide (0.5 μM for 4 hours, 0.2% O_2_) was also probed *in vitro*. Combined treatment also resulted in a statistically significant increase of cell killing in comparison to cellular treatment with evofosfamide and IR alone (Figure 4C).

### Induction of DNA damage and senescence in response to evofosfamide

**Figure 5:**
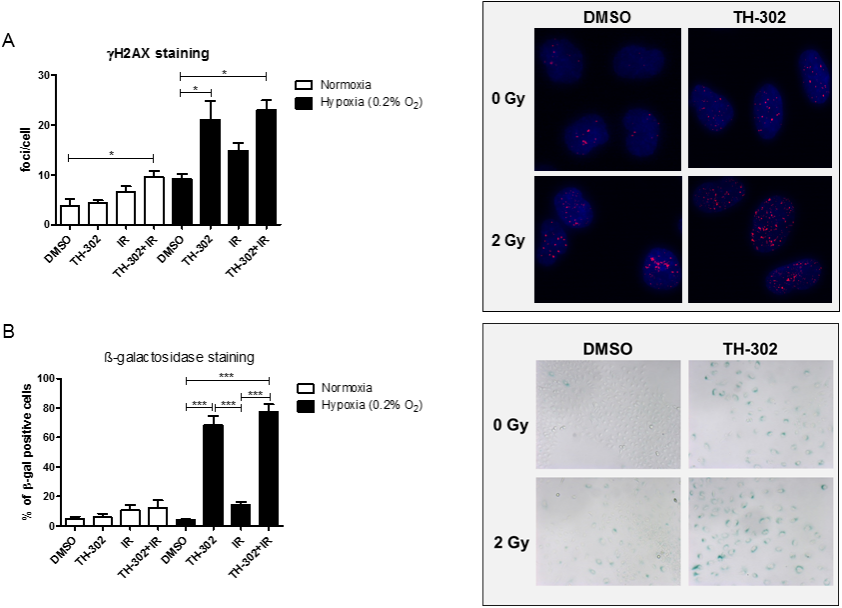
DNA damage in response to evofosfamide and irradiation **(A)** Residual γH2AX foci were analyzed in A549 cells treated for 4 hours with evofosfamide and irradiation with 2 Gy. Cells were analyzed 20 hours after irradiation at a magnification of 40x. **(B)** Induction of senescence (β-galactosidase staining) in response to the combined treatment in A549 cells. Cells were analyzed 72 hours after treatment at a magnification of 10x. At least 50 cells/condition were analyzed. Representative pictures are shown. Error bars represent SEM.

Activation of the prodrug evofosfamide results in the potent DNA-alkylating agent bromo isophosphoramide mustard and induces a strong DNA damage response. Residual DNA damage was determined by γH2AX foci detection in A549 cells on treatment with evofosfamide and IR alone and in combination. As expected, a high level of residual DNA-damage was present at the 24 hour time point on initial cellular incubation with evofosfamide for 4 hours under hypoxic but not under normoxic condition. Interestingly, combined treatment with irradiation (2 Gy) only resulted in a minimal but statistically not significant additional increase of residual DNA damage (Figure 5A). Lack of additional residual γH2AX foci in cells treated with both modalities does not correspond with the supra-additive cell killing by the combined treatment modality *in vitro* (see above Figure 4B, 4C). Similar results were obtained when DNA damage was probed on the level of residual 53BP1-foci (Supplementary Figure 5).

Senescence is a well-known mode of cell death induced upon treatment with alkylating agents [20]. A high percentage of β-galactosidase positive A549 cells was induced on treatment with evofosfamide under hypoxic conditions, which was further increased on combined treatment with IR (Figure 5B). These results demonstrate that evofosfamide alone induces a strong DNA damage response and senescence in lung carcinoma cells. The small increase in the number of senescent cells in response to evofosfamide in combination with IR corresponds in part with decreased clonogenicity of A549 cells in response to this combined treatment modality.

### Increased cell killing by evofosfamide in BRCA2-deficient ovarian carcinoma cells

**Figure 6:**
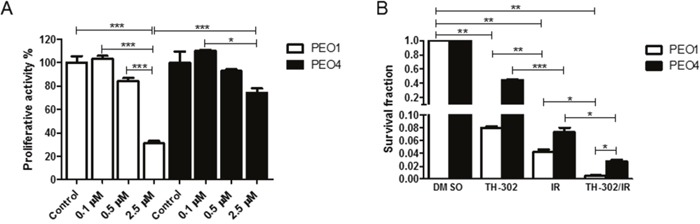
Treatment response of BRCA2-deficient (PEO1) and BRCA2-wild-type (PEO4) ovarian carcinoma cells to evofosfamide and irradiation **(A)** Cells were pre-incubated in hypoxia (0.2% O_2_) for 20 hours, followed by treatment with increasing concentrations of evofosfamide for 4 hours. The proliferative activity of reoxygenated cells was monitored over 72 hours. **(B)** Clonogenic cell survival assay of BRCA2 deficient (PEO1) and wild-type (PEO4) cells in response to evofosfamide (0.5 μM) and irradiation with 4 Gy. Cells were pre-incubated in hypoxia (0.2% O_2_) for 20 hours, followed by evofosfamide treatment under hypoxic conditions (4 hours), reoxygenation and irradiation with 4 Gy. Error bars represent SEM.

Evofosfamide-induced DNA damage requires homologous recombination for efficient DNA repair as previously shown in non-tumorigenic chinese hamster ovary cells [19]. To further investigate evofosfamide in combination with IR in tumorigenic cell lines and with defined HR-deficiency, proliferative activity and clonogenicity was determined in the BRCA2-wildtype PEO4 and the otherwise genetically identical BRCA2-deficient ovarian carcinoma cell line PEO1 [21]. As expected, BRCA2-deficient PEO1 cells were more sensitive to increasing concentrations of evofosfamide in comparison to BRCA2-wildtype PEO4 cells under hypoxic conditions (Figure 6A). Moreover, combined treatment with IR reduced clonogenic cell survival in PEO1 cells to a higher extent than in the BRCA2-wildtype counterpart cells (Figure 6B). The ratio of survival fractions in response to evofosfamide and evofosfamide in combination with IR was 25.5 and 17.9 for PEO1 and PEO4 cells, respectively. The ratio of survival fractions in response to IR and the combined treatment modality was 7.1 and 2.7 for PEO1 and PEO4, respectively. These results strongly indicate a superior appliance for this combined treatment modality in tumors with homologous recombination-deficiency.

## DISCUSSION

Evofosfamide is one of the most promising hypoxia-targeting agents currently tested in several clinical trials. Here we have investigated the combined treatment modality of evofosfamide with IR in a lung and a head&neck carcinoma model with a specific focus on multiple treatment regimens and schedulings. IR is known to target primarily well-oxygenated cells, while hypoxic cells are radiation-resistant. Therefore, the combined treatment modality of IR with evofosfamide is based on the promising rationale named biological cooperativity [5]. Both tumor models stained positive and to similar extent for the hypoxia-marker pimonidazole, however head&neck tumor xenografts were completely resistant to evofosfamide alone and when combined with IR, independent of the treatment scheduling. Subsequent expression studies revealed strongly reduced cytochrome P450 oxidoreductase (POR) levels in this tumor model. On the other hand A549 cells and A549 -derived tumor xenografts stained POR-positive and were highly evofosfamide-sensitive *in vitro* and *in vivo*.

Recently the dual combined treatment modality of IR with evofosfamide was shown to enhance the effect of radiotherapy in a rhabdomyosarcoma R1 and in a H460-derived non-small-cell lung cancer tumor model and as part of a trimodality therapy in sarcoma models with the anti-VEGF receptor-directed antibody DC101 [15, 22]. In both studies only single high doses of IR were applied and scheduling of evofosfamide with IR was not investigated.

We here tested three different treatment regimens (neoadjuvant, concomitant, adjuvant) in combination with fractionated and single high-dose IR in the lung adenocarcinoma tumor model. A more potent neoadjuvant and concomitant versus adjuvant scheduling of the combined treatment modality of evofosfamide with fractionated IR was identified, which might coincide with evofosfamide initially targeting the major tumor hypoxic burden followed by fractionated irradiation including partial reoxygenation of the remaining hypoxic areas.

On the other hand the more potent adjuvant scheduling of evofosfamide in combination with a single high dose of IR could be due to an (transient) increase of tumor hypoxia in response to single high doses of IR [23, 24]. Interestingly own experiments performed independently of this study also demonstrated an increase of tumor hypoxia over time in response to a single high dose of IR in this tumor model (Supplementary Figure 6). Similar to former studies with evofosfamide we only considered tumor growth delay as an endpoint and the hypoxic situation might vary in between different tumors. As such these schedulings will have to be carefully translated to the clinical situation.

Several studies on the activation of HAPs previously demonstrated that cytochrome P450 oxidoreductase is indispensable for several HAPs [18, 19, 25]. However, its relevance for the nitroimidazole mustard evofosfamide is less clear. Su et al. knocked-out POR in multiple tumor cell lines that resulted in resistance to several one-electron reductase substrates but not to evofosfamide, suggesting the existence of structure-dependent oxidoreductase redundancies [26]. On the other hand, Hunter et al. recently demonstrated reduced cytotoxicty of evofosfamide in head&neck carcinoma cells with shRNA-downregulated POR-expression [18]. More important, a heterogeneous POR expression status was retrospectively determined in head&neck squamous cell carcinoma patient samples, and suggests that POR might be a major predictive determinant for HAPs including evofosfamide [18]. Our own studies, which were performed on the cellular level and to our best knowledge for the first time also *in vivo*, strongly suggest that POR co-determines the potency of evofosfamide. As such we demonstrate minimal efficacy of evofosfamide *in vivo* in hypoxic tumor xenografts generated from a patient-derived head&neck carcinoma [27].

Only recently two phase III trials of evofosfamide in advanced soft-tissue sarcoma and in advanced pancreatic cancer in combination with doxorubicin and gemcitabine, respectively, did not meet their primary endpoints of improved overall survival (CancerNetwork Oncology, December 2015). Our *in vivo* data suggest the treatment combination of evofosfamide with radiotherapy is still of strong interest but requires detailed efficacy- and mechanistic-oriented studies towards a successful, personalized treatment approach. Our data demonstrate that both tumor hypoxia and the POR-status are strong co-determinant for the efficacy of evofosfamide. Thus, specific image- and gene expression-guided biomarker analysis to determine tumor hypoxia but also HAP-activation are required for optimized patient stratification. Serial analysis of serum factors specifically released in response to evofosfamide could represent a valid strategy to identify at an early stage evofosfamide-responsive tumors. We probed several angiogenesis- and hypoxia-related serum factors in response to evofosfamide. Interestingly only secretion of the placental growth factor (PlGF) was increased on treatment by evofosfamide under hypoxia and only by the evofosfamide-responsive and not by the evofosfamide-resistant carcinoma cells. Our *in vitro* data also corroborate that evofosfamide in combination with IR is more potent in BRCA2-deficient tumor cells than in their BRCA2-wildtype counterpart cells. Previous studies were only performed in genetically-defined CHO-cells and in combination with cisplatinum against tumor cells [18, 19].

Thus, it will be of highest interest to follow the results of the first clinical study by Larue and colleagues, testing evofosfamide with preoperative chemoradiotherapy in oesophageal adenocarcinoma patients [28]. This study includes repeated hypoxia PET imaging and blood sampling to determine hypoxia blood markers and could also incorporate testing of POR, PlGF and a putative homologous recombination-corrupted genetic background as part of their translational endpoints. Investigated dose levels of evofosfamide will range from 120 mg/m2 to 340 mg/m2 in this clinical study. However, it will be difficult to compare these dose levels with the drug concentrations applied in our animal study due to the differential route of drug administration and the differential drug metabolism between humans and mice. For comparative reasons we used similar concentrations of evofosfamide doses in our study as in the previous preclinical study by Peters et al. [15].

Overall our data demonstrate that evofosfamide with IR is a potent combined treatment modality against hypoxic tumors. Our preclinical data suggest that its efficacy on the clinical level could eventually be dependent on scheduling parameters and tumor type. Furthermore, several conditions linked to the individual geno- and phenotype of the tumor including the status of the HAP-activating oxidoreductases, DNA-damage repair machineries and the hypoxic burden have to be fulfilled, rendering this combined treatment modality highly potent towards a personalized treatment approach.

## MATERIALS AND METHODS

### Cell lines and compounds

The human non-small cell lung cancer (NSCLC) cell line A549 was obtained from ATCC and cultured in RPMI 1640 cell culture media supplemented with 10% FBS, glutamine (2 mM) and penicillin-streptomycin (100 U/ml-100 μg/ml). The head&neck squamous cell carcinoma (HNSCC) UT-SCC-14 cell line was a kind gift from Reidar Grénman (Turku University Hospital, Finland) and was maintained in DMEM, high glucose, NEAA, 10% FCS, 2 mM L-glutamine, 1% Penicillin/Streptomycin (100 U/ml-100 μg/ml) and 1 mM sodium pyruvate [27]. The ovarian cancer cells PEO4 and PEO1 were purchased from the Health Protection Agency Culture Collections (Salisbury, UK) and were kept in RPMI 1640 cell culture media supplemented with 10% FBS, glutamine (2 mM), sodium pyruvate (2 mM) and penicillin-streptomycin (100 U/ml-100 μg/ml). For normoxic conditions, cells were kept in a 5% CO_2_ incubator at 37°C, for hypoxic conditions, cells were kept in a 0.2% O_2_, 5% CO_2_, incubator (*In vivo*_2_ 300-Ruskinn; Hypoxia Incubator, Siemens) at 37°C. Evofosfamide was obtained from Merck KGaA.

### Cell proliferation and clonogenic cell survival assay

The proliferative activities of tumor cells were assessed in 96-well plates with the colorimetric alamarBlue assay (Invitrogen). Cells were preincubated under hypoxia and treated for the indicated time intervals and concentrations of evofosfamide in either normoxic (21% O_2_) or hypoxic (0.2% O_2_) conditions. Clonogenic cell survival was determined by the ability of single cells to form colonies *in vitro* as described before [29]. Cells were treated with 0.5 μM of evofosfamide for 4 hours in either normoxic (21% O_2_) or hypoxic (0.2% O_2_) conditions or as described in the figure legend. Thereafter cells were reoxygenated and irradiated with increased doses of IR and trypsinized. To probe the adjuvant scheduling, cells were first irradiated with 2 Gy, followed by addition of evofosfamide and incubation of cells in either normoxic or hypoxic conditions for 4 hours. Single cell suspensions were seeded into 10 cm-petri dishes. The number of plated cells per dish was adjusted to obtain approx. 50-100 colonies under all experimental conditions. After colony formation (depending on cell lines, approx. 14 days), colonies were fixed (methanol/acetic acid; 3:1) and stained with crystal violet (2%). Colonies (containing > 50 cells) were then counted manually.

### Tumor xenografts and application of treatment regimes

A549 lung carcinoma and UT-SCC-14 head&neck squamous cell carcinoma cells were subcutaneously injected on the back of four week old, female CD1 athymic nude mice (Charles River). Tumor volumes were determined from caliper measurements of tumor length (L) and width (l) according to the formula (L x l^2^)/2. Treatment was initiated when tumors reached a volume of 300 mm^3^ +/− 10%. Tumors were sham-irradiated or irradiated using a customized shielding device with either a fractionated (3×2 Gy) or a single high dose regimen (1×10 Gy) using an Xstrahl 200 kV X-ray unit at 1 Gy/min. Evofosfamide (50 mg/kg in saline) or saline was administered i.p. Q3Dx5. Three treatment regimens were investigated: neoadjuvant, with evofosfamide on days 1-12 followed by either a fractionated or a high dose regimen; concomitant, with evofosfamide on days 1-12 and IR on days 5-7 (fractionated) or day 6 (high dose); adjuvant, IR on days 0-2 (fractionated) or day 0 (high dose) followed by treatment with evofosfamide. All *in vivo* experiments were performed according to the guidelines for the welfare and use of animals of the Veterinäramt Kanton Zürich, Switzerland.

### Bio-plex multiplex assay

A549 lung carcinoma and UT-SCC-14 head&neck squamous cell carcinoma cells were seeded in 6-well plates at the density of 150-200′000 cells/well in DMEM medium (high glucose, NEAA, 10% FCS, 2 mM L-glutamine, 1% Penicillin/Streptomycin and 1 mM sodium pyruvate). Cells were irradiated with 5 Gy followed by addition of evofosfamide (0.5; 1 μM) and placed in either normoxic (21% O_2_) or hypoxic conditions (0.2% O_2_). After 24 hour incubation, conditioned medium was collected, filtered through an 0.45 μM filter and stored at -20°C until analysis. A customized Bio-plex Biomarker Cancer Panel assay was performed with undiluted conditioned medium samples according to the manufacturer protocol (Bio-Rad). Obtained concentrations of measured samples (pg/ml) were normalized to the cell number and are shown as fold induction relative to the determined concentrations derived from normoxic untreated control samples.

### Western blotting and immunohistochemistry

For western blot analysis, A549 and UT-SCC-14 cells were incubated in either normoxic (21% O_2_) or hypoxic conditions (0.2% O_2_) for 24 hours, followed by lysis in RIPA buffer (Sigma) and SDS-PAGE. Membranes were incubated with primary anti-Cytochrome P450 Reductase (POR/CYPOR) antibodies (Santa Cruz Biotechnology (G-5): sc-25263; 1:100) and mouse monoclonal anti–β-actin antibody (Sigma Aldrich, #A5441, 1:1000), followed by secondary anti-mouse ECL IgG HRP-linked (GE Healthcare, NA931V, 1:2000). Immunohistological endpoints were analyzed on paraffin-embedded blocks of A549 and UT-SCC-14-derived tumor xenografts using anti-POR (Invitrogen, PA5-27326; 1:100) and anti-pimonidazole (Hypoxyprobe, HP1-1000, 1:100) antibodies.

### γH2AX and 53BP1 foci staining

A549 cells were preincubated in hypoxic conditions (0.2% O_2_) for 20 hours, followed by treatment with evofosfamide for 4 hours under hypoxic conditions, reoxygenation and irradiation with 2 Gy. 20 hours after irradiation, cells were washed twice with PBS, fixed with 4% formaldehyde/PBS for 10 min, and washed with PBS (4 × 5 min). Cells were then permeabilized for 5 min with 0.2% ice cold Triton-X-100/PBS, blocked for at least 20 min with 1% BSA, followed by 1 hour incubation with the rabbit monoclonal anti-H2AX-pSer139 (1:100, Abcam, Cambridge, UK) or the rabbit polyclonal anti-53BP1 (1:200, Cell Signaling, Boston MA, USA) primary antibodies, diluted in 1% BSA/PBS. After washing with 1% BSA/PBS (3 × 15 min), cells were incubated with the appropriate secondary antibody diluted 1:1000 (Alexa-488), washed with 1% BSA/PBS (2 × 10 min) followed by PBS (1 × 10 min) and incubated with DAPI/Methanol (1 μg/ml) for 3 min, before fixation with Dako Fluorescent Mounting Medium (Dako, North America). Images were taken using a Leica DM 5500 microscope at a magnification of 40x and quantified using FociCounter software. At least 50 cells/condition were analyzed.

### Analysis of cellular senescence

A549 cells were preincubated in hypoxic conditions (0.2% O_2_) for 20 hours, followed by treatment with evofosfamide for 4 hours under hypoxic conditions, reoxygenation and IR with 2 Gy. 72 hours after irradiation, cells were stained for β-galactosidase using the Senescence β-Galactosidase Staining Kit (Cell Signaling, #9860): cells were washed with PBS and fixed (with 2% formaldehyde, 0.2% glutaraldehyde in PBS) for 15 min at room temperature. Cells were washed twice with PBS and stained with 40 mM citric acid/sodium phosphate (pH 6.0), 0.15 M NaCl, 2 mM MgCl_2_, 5 mM potassium ferrocyanide, 5 mM potassium ferricyanide, 1 mg/ml X-gal (5-bromo-4-chloro-3-indolyl-βD-galactopyranoside) for 24 hours at 37°C. β-galactosidase-positive cells were counted in at least 3 randomly chosen visual fields at a magnification of 10x in each treatment setup.

### Short interfering RNA treatment

A549 cells were transfected with POR-directed siRNA (Santa Cruz Biotechnology) for 24h using Lipofectamine RNAiMAX (Invitrogen). Cells were preincubated under hypoxia (0.2% O_2_) for 20 hours, followed by incubation with evofosfamide for 4 hours under hypoxia and reoxygenation.

### Statistical analysis

For *in vivo* treatment response to evofosfamide with fractionated and single high dose irradiation, the mean slopes of tumor growth curves for individual animals (day 0-15) were calculated and analyzed by one way ANOVA with Tukey post test using the GraphPad software. To calculate statistical significance between two or more groups of variables in *in vitro* experiments, either unpaired t-test or ANOVA with Tukey post test was used, respectively. P values <0.05 were considered significant. For all experiments, *P<0.05, **P<0.01, ***P<0.001.

## SUPPLEMENTARY MATERIALS FIGURES AND TABLES


