# Supplementary material for: The hypoxia-activated prodrug evofosfamide in combination with multiple regimens of radiotherapy
Source: Oncotarget. 2017 Feb 28;8(14):23702–12. doi: 10.18632/oncotarget.15784 (PMC5410338; doi:10.18632/oncotarget.15784)
Supplement: Supplementary file 1 [file oncotarget-08-23702-s001.pdf]

The hypoxia-activated prodrug evofosfamide in combination with multiple regimens of radiotherapy

SUPPLEMENTARY DATA

REFERENCE

1. Orlowski K, Bley CR, Zimmermann M, Van V, Hug D, Soltermann A, et al. Dynamics of Tumor Hypoxia in Response to Patupilone and Ionizing Radiation. Plos One. 2012; 7.

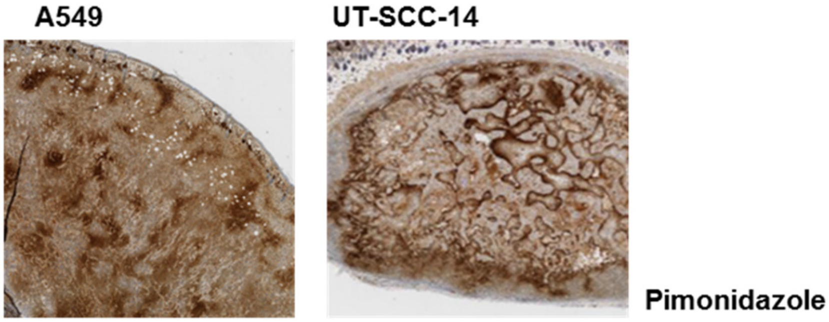

Supplementary Figure 1: Tumor hypoxia in A549 and UT-SCC-14-derived tumor xenografts. Staining of A549 (left) and UT-SCC-14 (right)-derived tumor xenografts for pimonidazole uptake. Pimonidazole was injected 1 hour (i.p.) prior euthanasia and tumor extraction.

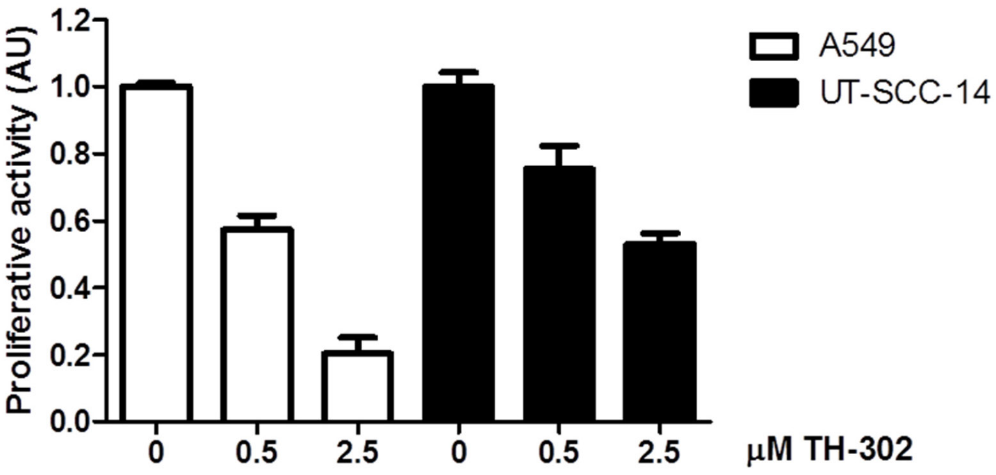

Supplementary Figure 2: Differential sensitivity of A549 and UT-SCC-14 cells to evofosfamide *in vitro*. Proliferative activity of A549 and UT-SCC-14 cells 72 hours after evofosfamide treatment. Cells were preincubated under hypoxia (0.2% O_2) for 20 hours, followed by treatment with increasing concentrations of evofosfamide for 4 hours, followed by reoxygenation. Error bars represent SEM.

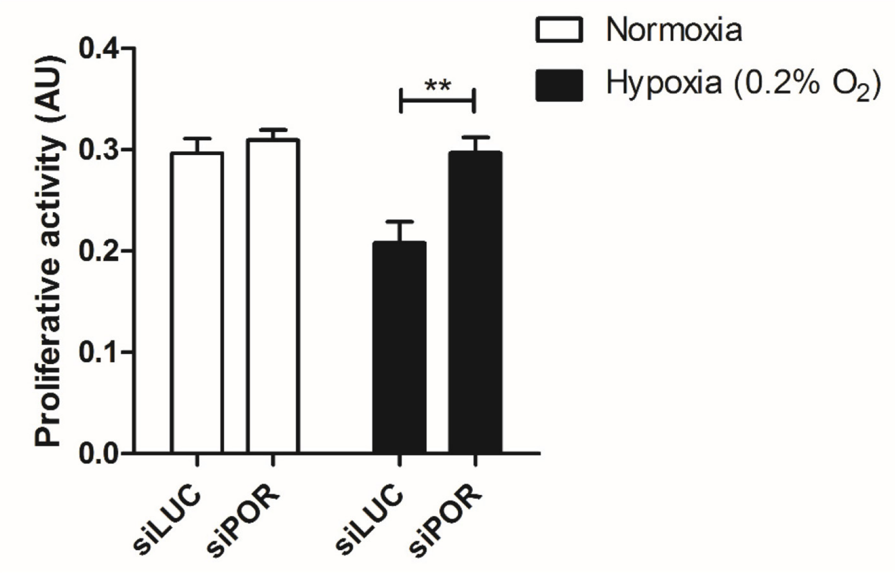

Supplementary Figure 3: Effect of POR downregulation on A549 cell sensitivity towards evofosfamide *in vitro*. Proliferative activity of siLUC and siPOR-pretreated A549 cells 48 hours after evofosfamide treatment. Cells were preincubated under hypoxia (0.2% O_2) for 20 hours, followed by treatment with evofosfamide (0.5µM) for 4 hours and reoxygenation. Error bars represent SEM.

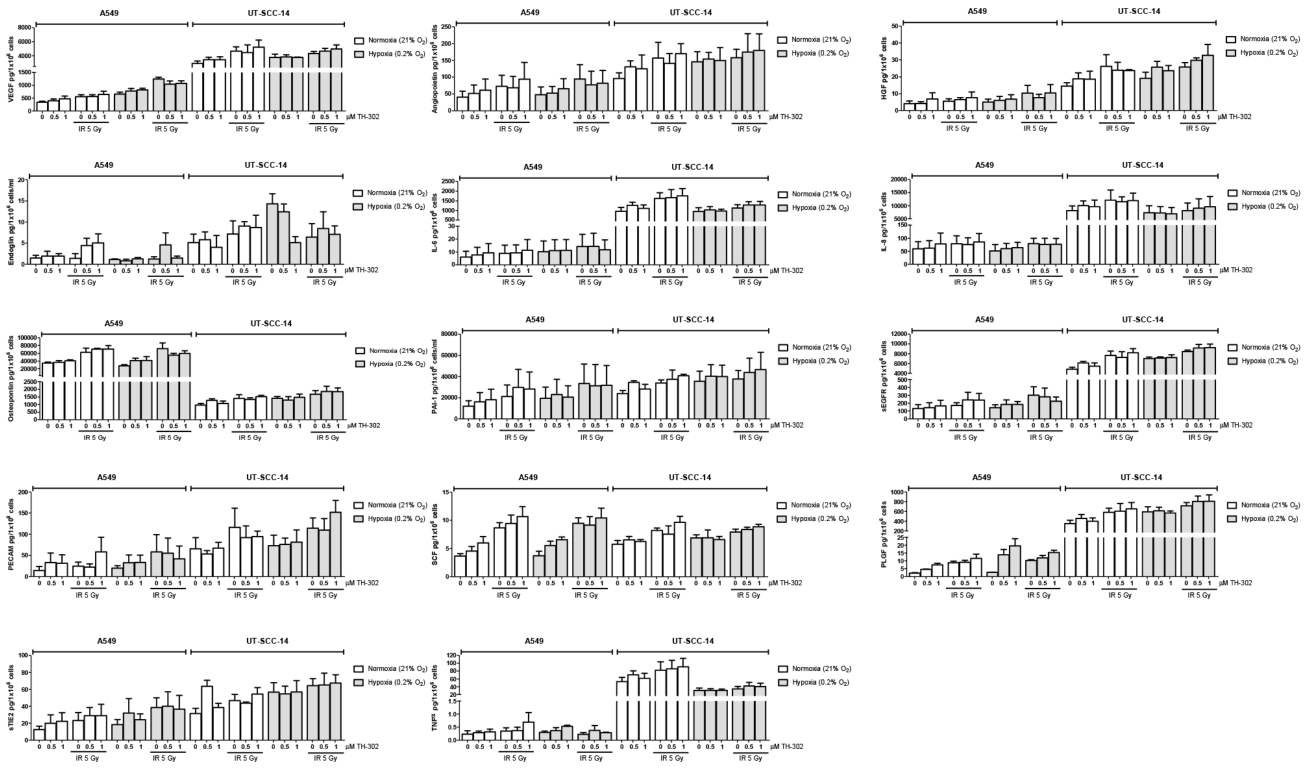

Supplementary Figure 4: Analysis of secreted factors in response to evofosfamide and irradiation. Raw data of Bioplex analysis of secreted factors in A549 and UT-SCC-14 cells in reponse to evofosfamide (0.5, 1 µM) and irradiation (5 Gy) under normoxic (21% O_2) and hypoxic conditions (0.2% O_2). Error bars represent SEM.

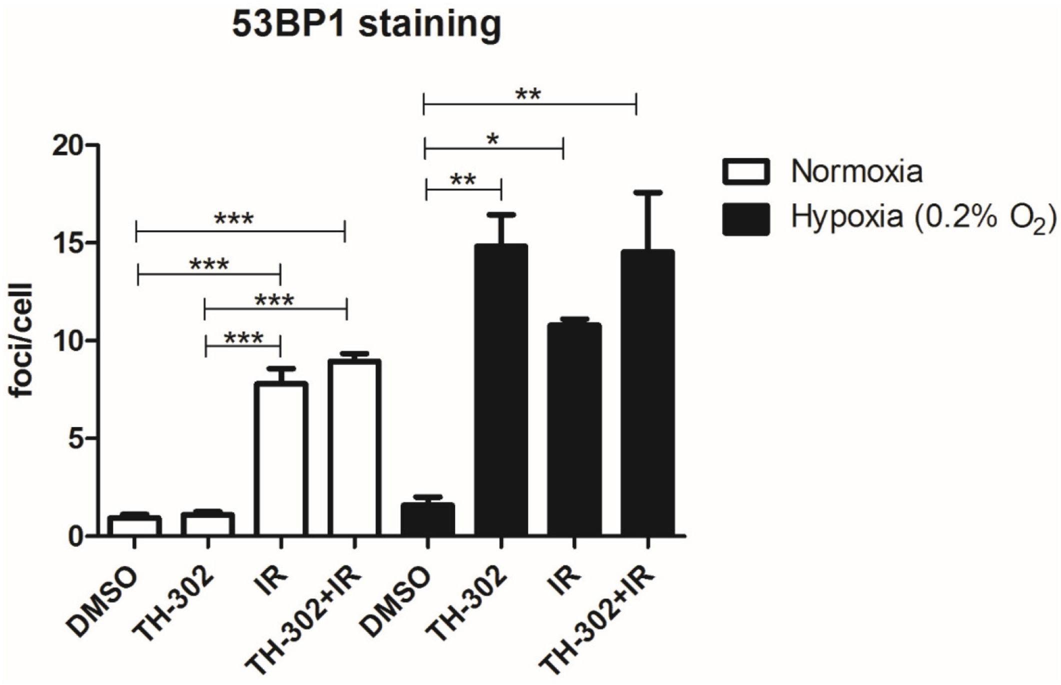

Supplementary Figure 5: DNA damage in response to evofosfamide and irradiation. 53BP1 foci were analyzed in A549 cells treated for 4 hours with evofosfamide and irradiation with 2 Gy. Cells were analyzed 20 hours after irradiation. Error bars represent SEM.

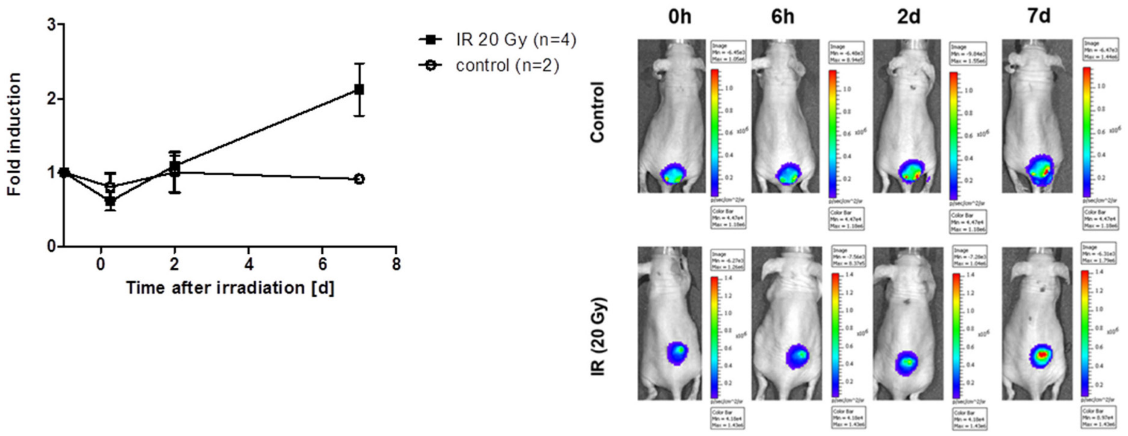

Supplementary Figure 6: Luciferase-activity of A549 ODD-Luc-derived tumor xenografts untreated and treated with single high-dose IR (20 Gy). Representative *in vivo* bioimages of control and irradiated mice. Measurements were performed as described in [1]. Data are shown as fold induction of the total flux normalized to the tumor volume at the indicated time point. Error bars represent SEM.
